# Robust phenotype prediction from gene expression data using differential shrinkage of co-regulated genes

**DOI:** 10.1038/s41598-018-19635-0

**Published:** 2018-01-19

**Authors:** Kourosh Zarringhalam, David Degras, Christoph Brockel, Daniel Ziemek

**Affiliations:** 1 Department of Mathematics, University of Massachusetts Boston, Boston, MA 02125 USA; 2 Computational Sciences, Pfizer Worldwide Research & Development, Cambridge, MA 02139 USA

## Abstract

Discovery of robust diagnostic or prognostic biomarkers is a key to optimizing therapeutic benefit for select patient cohorts - an idea commonly referred to as *precision medicine*. Most discovery studies to derive such markers from high-dimensional transcriptomics datasets are weakly powered with sample sizes in the tens of patients. Therefore, highly regularized statistical approaches are essential to making generalizable predictions. At the same time, prior knowledge-driven approaches have been successfully applied to the manual interpretation of high-dimensional transcriptomics datasets. In this work, we assess the impact of combining two orthogonal approaches for the discovery of biomarker signatures, namely (1) well-known lasso-based regression approaches and its more recent derivative, the group lasso, and (2) the discovery of significant upstream regulators in literature-derived biological networks. Our method integrates both approaches in a *weighted group-lasso* model and differentially weights gene sets based on inferred active regulatory mechanism. Using nested cross-validation as well as independent clinical datasets, we demonstrate that our approach leads to increased accuracy and generalizable results. We implement our approach in a computationally efficient, user-friendly R package called *creNET*. The package can be downloaded at https://github.com/kouroshz/creNethttps://github.com/kouroshz/creNet and is accompanied by a parsed version of the STRING DB data base.

## Introduction

Discovery of robust diagnostic or prognostic biomarkers is a key to optimizing therapeutic benefit for select patient cohorts - an idea commonly referred to as *precision medicine*^1^. Biomarkers are usually discovered on a small specific sub-sample of patients, e.g. from a small set of clinics in a particular country, but will be applied in a much larger and more diverse patient population. Ultimately, the quality of a biomarker hinges on its predictive performance in the general patient population.

Most discovery studies to derive markers from high-dimensional transcriptomics datasets are weakly powered with sample sizes in the tens of patients. Even clinical trials with accompanying omics measurements usually enlist far fewer than 100 patients per study arm. Methodologically, we are seeking a trained classifier that can predict a future phenotypic outcome, e.g. kidney rejection after a transplant or response to a drug, based on high-dimensional transcriptomics data at baseline. A particular focus of this study will be the evaluation in terms of *independent* datasets with clinically similar characteristics to understand the risk of overfitting based on small discovery studies^2^.

Given the high dimensionality of transcriptomics data, regularized statistical approaches are essential to making robust predictions^3^. Regularized classification methods such as ${\ell }_{1}$ regularized logistic regression (also known as lasso)^4^ or its variations^5,6^ are very popular machine-learning techniques for addressing the high dimensionality of the datasets. Automatic variable selection as well as sparsity and high interpretability of the results are some of the reasons of the popularity of these methods. In recent years, a new class of penalized regression methods that use a penalty term for group-wise shrinkage of covariates has garnered considerable interest^7–10^. This new type of penalization is widely referred to as the ‘group-lasso’ and was originally developed in^7^ for linear regression in the case where there is no overlap between pre-defined groups of covariates. The penalty was later extended to the case of logistic regression^8^. In^11^, the authors formulated the problem in a unified approach that contains linear regression, logistic regression and support vector machines as sub-cases. The model was generalized in^9^ to include an additional within group sparsity penalty term. Finally, in^10^, the authors introduced a variation of the group-lasso norm, called ‘overlap-norm’ to extend the method to cases when there is overlap between groups.

Another approach to avoid overfitting consists in incorporating prior biological knowledge into the classification procedure. This type of approach has been successfully applied to the manual interpretation of high-dimensional datasets. Gene set and pathway enrichment methods^12^ are a mainstay of expert-driven analysis of transcriptomics data. Commercial tools such as Qiagen’s IPA^13^ are used to discover biological insights in numerous biomedical publications (https://www.qiagenbioinformatics.com/). A more recent approach is the inference of upstream causal regulators based on a literature-derived network to guide the researcher to proximal or distal causes of the observed gene expression changes^13–15^. Recently, we presented an improved method for discovering upstream regulators based on a generalization of Fisher’s exact test that works well with a given mixed causal/non-causal gene regulatory network and a set of differentially expressed genes^16^.

In this work, we assess the impact of combining two orthogonal approaches for the discovery and independent validation of biomarker signatures, namely (1) well-known lasso-based regression approaches and its more recent derivative, the group lasso, and (2) the discovery of significant upstream regulators in literature-derived biological networks. Our method integrates both approaches in a *weighted group-lasso* model, specifically designed to predict a phenotype using baseline gene expression data. In particular, our method differentially weights gene sets based on inferred ‘active regulatory mechanism’. This shrinkage can result in optimal sparsity and model robustness in scenarios where the differences between patient subpopulations are governed by differentially regulated pathways as opposed to differences at the single gene level, which is often the case in complex diseases. We demonstrate this advantage using simulation studies as well as real datasets from independent clinical trials.

There has been related work to integrate different data modalities or prior knowledge into the classification procedure. Cun *et al*.^17^ gives a recent review. In particular, PARADIGM^18^ utilizes a probabilistic approach to combine multiple sources of biological data to identify significant pathways in cancer patients. However, PARADIGM is not designed for response prediction using baseline gene expression data in clinical trials. Several other graph-based approaches for phenotype prediction have been developed^19–23^. For instance, authors in^22^ use a penalty term based on the Laplacian of the gene interaction graph that will impose similar weights to genes that are closer together in the network. This penalty is also used in logistic regression setting for classification and pathway association problems^24^. Although these methods generally have acceptable performance, there are many instances where they fail to generalize to independent datasets^23^.

Our method differs from other methods that integrate network data into the classification process in several key points, namely (1) we achieve dimension reduction by identifying active regulators and their downstream genes in a biologically meaningful way, (2) we incorporate a weighted shrinkage of co-regulated gene sets that is proportional to the inferred relevance of the set to the phenotype, (3) we achieve optimal model sparsity through simultaneous regularization of co-regulated gene sets as well as individual genes within each sets while taking the overlap between the biological processes into consideration, (4) in addition to predictive gene signatures, our algorithm outputs active regulators that are predictive of disease progression or response to drug treatment, which increases the biological interpretability of the identified markers.

We provide an efficient an user friendly R package, *creNET* accompanied with processed gene-regulatory network obtained from STRING-DB^25^ and command line R scripts. The package can be downloaded at https://github.com/kouroshz/creNethttps://github.com/kouroshz/creNet.

## Methods

The starting point of our method is a set of per-patient baseline gene expression measurements as well as a gene regulatory network. Our method, *creNET*, consists of two integral parts, (1) the discovery of significant upstream regulators and accompanying weights from literature-derived biological networks, (2) the efficient incorporation of this information into a group lasso procedure.

### Upstream regulator detection

**Figure 1:**
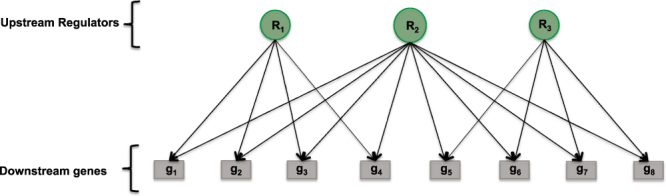
Figure shows a schematic representation of gene sets defined by a regulatory network. The regulators *R*_1_, *R*_2_ and *R*_3_ group the downstream genes into gene sets *g*^(1)^ = {*g*_1_, *g*_2_, *g*_3_, *g*_4_}, *g*^(2)^ = {*g*_1_, *g*_2_, *g*_3_, *g*_4_, *g*_5_, *g*_6_, *g*_7_, *g*_8_} and *g*^(3)^ = {*g*_5_, *g*_6_, *g*_7_, *g*_8_}. Note that the gene sets may overlap.

We recently introduced a new ‘active gene regulator’ detection method that is suitable for use with public protein-protein, protein-gene interaction networks with a mix of signed and unsigned causal edges. In this context, let *G* = (*V*, *E*) be a given gene regulatory networks, where *V* is the set of vertices, typically consisting of Transcription Factors, Proteins, miRNAs, Compounds, etc., and *E* is the set of edges. The existence of an edge between a source node *v* and a target node *g* (*v* → *g*) implies that *v* regulates *g*. For the purpose of this paper we assume that the target nodes *g* are genes. If the edge between *v* and *g* is signed, then the direction of the regulation is known with + indicating ‘*v* up-regulates *g*’, and − indicating ‘*v* down-regulates *g*’. The network groups the gene into classes (or gene sets) according to the connections of ‘upstream regulators’ to downstream genes. Figure 1 shows a schematic plot of gene sets defined by a gene regulatory network.

The method outputs likely upstream regulators based on differentially expressed genes between two conditions. It quantifies the statistical significance of each potential regulator with respect to a null model of random assignment of differential expression status to genes in the network. If edges in the network are signed, the direction of regulation will influence the test statistic and, thus, give more credence to upstream regulators that respect the regulation pattern of their downstream genes. For more details, we refer to the original publication^16^. The significance estimates for each potential regulator will serve as weights for the corresponding gene set in our creNET procedure.

There are many academic^25,26^ and commercial (Ingenuity: www.ingenuity.com and Selventa: www.selventa.com) sources of biological networks. In our current implementation, we utilize a commercially curated collection of interactions that consists of approximately 87,140 nodes and 671,371 edges, where each edge is supported by an experiment reported in the literature. We refer to this network as *Causal Reasoning Engine Knowledge Base* or *creKB* for short. However, our method also works with other types of network. In particular, the released package comes with version 10 of the STRING DB data base^25^ and we provide equivalent figures for all results based on the STRING DB in the Additional File 1. We refer to this network as *publicKB*.

### Shrinkage of co-regulated gene sets

In order to integrate information on regulatory interactions and co-regulated gene sets into the classification In order to integrate information on regulatory interactions and co-regulated gene sets into the classification task, we utilize group-wise regularization of covariates as follows. Given a set of training data ${\{({y}_{i},{{\bf{x}}}_{i})\}}_{i=1}^{n}$, where ${{\bf{x}}}_{i}\in {{\mathbb{R}}}^{p}$ is the vector of normalized gene expression values for the *i*-th patient and *y*_*i*_ ∈ {−1, 1} is a binary response, we construct an *n* × *p* design matrix *X* and a binary response vector *y* = (*y*_1_, …, *y*_*n*_)^*T*^. The classification problem can be quite generally stated as an optimization problem of the form:

1 $$\mathop{{\rm{argmin}}}\limits_{\beta }\frac{1}{n}\sum _{i=1}^{n}\Phi ({y}_{i},{\beta }^{T}{{\bf{x}}}_{i})+\lambda g(\beta ),$$

where Φ is a loss function that links the covariates to the response via the linear function *β*^*T*^**x**_*i*_, *g* is a penalty term and *λ* is a tuning parameter^11^. Here $\beta ={({\beta }_{1},\cdots {\beta }_{p})}^{T}\in {{\mathbb{R}}}^{p}$ is the vector of unknown coefficients. Several choices of loss functions such as linear regression and Support Vector Machines (SVMs) can be formulated as above. For our purpose, we utilize logistic regression:

2 $$\Phi ({y}_{i},{\beta }^{T}{{\bf{x}}}_{i})=\,\mathrm{log}(\frac{1}{1+{e}^{{y}_{i}{\beta }^{T}{{\bf{x}}}_{i}}}).$$

For ease of presentation, we do not consider the intercept term *β*_0_ separately; addition of this term to the loss function can be trivially achieved by adding an extra column of 1’s at the beginning of the design matrix *X*.

The group lasso penalty introduced in^7^ provides a natural way for simultaneous shrinkage of co-regulated gene sets. Assume that the gene regulatory network categorizes the genes into *K* (possibly overlapping) groups ${\mathscr{G}}=\{{g}^{(1)},{g}^{(2)},\cdots {g}^{(K)}\}$ (Fig. 1). Moreover, let ${\beta }^{(\ell )}$ be the vector obtained from *β* by keeping all components of *β* that correspond to the group ${g}^{(\ell )}$ identical and setting all other components to zero. Note that ${\rm{supp}}({\beta }^{(\ell )})\subset {g}^{(\ell )}$, where ${\rm{supp}}({\beta }^{(\ell )})$ denotes the support of ${\beta }^{(\ell )}$. The group-lasso norm is then defined by

3 $$||\beta |{|}_{{\mathscr{G}}}=\sum _{g\in {\mathscr{G}}}{\Vert {\beta }^{(g)}\Vert }_{2}.$$

This penalty is in fact the ${\ell }_{1}$ norm of the ${\ell }_{2}$ norm of the group coefficient vectors (*β*^(1)^, …*β*^(*K*)^). This quantity is non-differentiable at ${\beta }^{(\ell )}\mathrm{=0}$ and hence acts as a regular ${\ell }_{1}$ penalty, but sets the coefficient of the entire gene group to 0. If some of the groups overlap, the quantity (3) is still a norm whose unit ball has singularities when some of the ${\beta }^{(\ell )}\mathrm{=0}$. In this case, it is straightforward to show that

4 $${\rm{supp}}(\beta )\subset {(\mathop{\cup }\limits_{g\in {{\mathscr{G}}}_{0}}g)}^{c},$$

where ${{\mathscr{G}}}_{0}\subset {\mathscr{G}}$ denotes the set of groups such that *β*^(*g*)^ = 0 and the exponent, *c*, denotes the set complement.

An important implication of the above equation is that in presence of overlapping groups, the group-lasso penalty will result is sparse solutions where the active (i.e., non-zero) groups may include genes with zero coefficients. For instance if groups ${g}^{(\ell )}$ and *g*^(*k*)^ have covariates *x*_1_, …*x*_*m*_ in common, and if ${g}^{(\ell )}$ is set to zero, the coefficients of these same covariates in *g*^(*k*)^ will also be set to zero. This is not a desirable effect. In particular, consider a situation where the covariates *x*_1_, …*x*_*m*_ weakly correlate with the phenotype and no other covariate in group ${g}^{(\ell )}$ is significant, so the entire group is set to zero. However, same covariate, when considered with the remaining covariates in group *g*^(*k*)^ may make the entire group significant and hence should remain in the model. As most regulatory biological networks will have overlapping groups, we are interested in a penalty such that the support of *β* is a union of groups, i.e., the opposite of the above equation. In^10^, the authors introduced an overlapping group-penalty defined by

5 $$||\beta |{|}_{{\mathscr{O}}}=\mathop{\min }\limits_{\begin{array}{c}{v}^{(1)},\cdots {v}^{(K)}\in {{\mathbb{R}}}^{p}\\ \beta =\sum {v}^{(\ell )}\end{array}}\sum _{{g}^{(\ell )}\in {\mathscr{G}}}{\Vert {v}^{(\ell )}\Vert }_{2},$$

where ${\{{v}^{(\ell )}\}}_{\ell =1}^{K}$ is a partition of *β* such that ${\rm{supp}}({v}^{(\ell )})\subset {g}^{(\ell )}$ for all $\ell$. This penalty has the desirable property that if a group is active (non-zero), all of the down-stream genes will have a non-zero coefficient, even if the genes are shared by inactive (i.e., zero) groups, i.e.,

6 $${\rm{supp}}(\beta )\subset \mathop{\cup }\limits_{g\in {{\mathscr{G}}}_{1}}g,$$

where ${{\mathscr{G}}}_{1}\subset {\mathscr{G}}$ denotes the set of groups such that *v*^(*g*)^ ≠ 0. In order to achieve within-group sparsity, an ${\ell }_{1}$ penalty of covariates can be added resulting in a penalty of the form

7 $$\Psi (\beta )=\alpha {\Vert \beta \Vert }_{{\mathscr{O}}}+\mathrm{(1}-\alpha ){\Vert \beta \Vert }_{1},$$

in the objective function (1), where *α* ∈ [0,1] is a tradeoff parameter between group-wise and single gene penalties.

The calculation of the overlapping group norm (5) based on its direct definition amounts to a nonsmooth convex optimization problem under linear constraints, which is in general highly nontrivial. To facilitate computations (at the expense of artificially increasing the size of the data), a simple duplication device can be used to replace the overlapping group norm with an equivalent non-overlapping group-lasso norm via^10^. More precisely, let $M={\sum }_{j=1}^{K}\#({g}^{(j)})$ be the total number of genes, taking their multiplicity into consideration. For each group of co-regulated genes *g*^(*j*)^, let *X*^(*j*)^ be the *n* × #(*g*^(*j*)^) slice of the gene expression matrix *X* that contains the genes regulated by the *j*-th regulator. Let $\tilde{X}=[{X}^{(1)},\cdots ,{X}^{(K)}]$ be the concatenation (duplication) matrix of size *n* × *M* and let *x*_*i*_, *i* = 1, …, *n* denote the rows of $\tilde{X}$. For a vector $\tilde{\beta }\in {{\mathbb{R}}}^{M}$, we write $\tilde{\beta }=({\tilde{v}}^{\mathrm{(1)}},\cdots \,{\tilde{v}}^{(K)})$ with ${\tilde{v}}^{(j)}\in {{\mathbb{R}}}^{\#({v}^{(j)})}$, *j* = 1,…, *K*. To each ${\tilde{v}}^{(j)}$, we associate a vector ${v}^{(j)}=(0,\cdots ,0,{\tilde{v}}^{(j)},0,\cdots ,0)\in {{\mathbb{R}}}^{p}$ such that sup (*v*^(*j*)^)∈*g*^(*j*)^. The classification can now be performed on the duplicated gene expression matrix $\tilde{X}$ with regression coefficients $\tilde{\beta }\in {{\mathbb{R}}}^{M}$ and non-overlapping group norm $||\tilde{\beta }{||}_{{\mathscr{G}}}={\sum }_{j\mathrm{=1}}^{K}||{v}^{(j)}{||}_{2}$. A formal derivation of the equivalence between overlapping group penalty and duplicated non-overlapping group-penalty is presented in the Additional File 1.

### Differential shrinkage of gene sets based on the significance of upstream regulators

The penalty term $||\tilde{\beta }|{|}_{{\mathscr{G}}}={\sum }_{j=1}^{K}||{v}^{(j)}|{|}_{2}$ can be modified to include weights as $||\tilde{\beta }|{|}_{{\mathscr{G}}}={\sum }_{j=1}^{K}{w}_{j}||{v}^{(j)}|{|}_{2}$. In the original introduction of the group-lasso norm and its subsequent developments, the weights *w*_*j*_ are generally set to #(*g*^(*j*)^)^1/2^, i.e., the square root of the number of genes in the *j*-th regulation group^7^. While this choice seem reasonable, it may have unintended consequences. Indeed these weights may cause larger groups to be set to zero and only let smaller groups survive, regardless of the biological importance of the regulator under consideration. For instance, an important regulator in Kidney Rejection (one of the diseases that we study in this paper) is IFNG. This regulator corresponds to a very large gene set that is heavily penalized under the square-root penalty and is not retained in the fitted model.

Intuitively, gene sets should be penalized based on the relevance of the set to the phenotype. The inferred significance of upstream regulators of the gene sets provides a natural way to achieve this. Below, we present four sets of weights, designed to systematically assess the impact of differential shrinkage on model performance and robustness.

1. *unweighted*: No weights are applied to groups. This weighting is equivalent to *unweighted SGL*^7,9^.
2. *sqrt-weighted*: Square root of the number of genes in the groups. This weighting is equivalent to *weighted SGL*^7,9^.
3. *regulator-weighted*: Inferred *p*-values (of active upstream regulators of the gene group).
4. *regulator-sqrt-weighted*: Inferred *p*-values times the square root of the group size.

### creNET: implementation

For the fitting part of the algorithm, we modified the source C++ implementation of the fitting process from the R package *SGL*^9^. As part of the modification, we added the inclusion of arbitrary weights into the fitting process. We then developed an independent R package called *creNET* that includes several auxiliary and utility functions. These include functions for network and data processing, data normalization, calculation of an optimal path of *λ* values^9^, data filtering and weight generation, goodness-of-fit measures such as AUC, nested cross-validation, independent training and testing, and plotting utilities. Moreover, we provide a parsed version of the STRING DB database as well as scripts for running various tests. The package is available to download from github at https://github.com/kouroshz/creNet. See Additional File 1 for additional algorithmic details.

### Evaluation procedure

We compared the proposed methods to well-established benchmark methods using artificial as well as empirical clinical data sets. Our numerical experiments were designed to test various aspects of our model including (1) the advantage of group-based vs. single gene shrinkage, (2) the importance of gene sets defined by upstream regulatory interactions, (3) the impact of weighted shrinkage based on the inferred activity of upstream regulators, (4) the generalizability of trained predictive models to new datasets and (5) the biological relevance of the significant predictors to the phenotype. We used simulation and randomization studies to assess items 1 and 2, while 3 and 4 were tested on real clinical trial datasets by cross-validation as well as independent training and testing. An advantage of our method is that in addition to the class labels, it outputs gene sets and upstream regulators with significant discriminatory power. We studied the biological relevance of these regulators to the phenotype in each of our datasets to address item 5.

Table 1 gives an overview of the benchmark methods. Ridge (#1) and lasso (#2) logistic regressions are popular approaches for phenotypic prediction^27^. To assess the impact of feature selection by the upstream regulator detection method, we also defined the Filtered Gene lasso (#3). In this method, we simply restrict the input transcriptomics data to genes that are regulated by active upstream regulators. Finally, we consider the four group lasso-based approaches defined in the previous section. All parameters for detection of upstream regulators are the same across methods. For a fair comparison between methods, input train and test gene expression data are filtered to include only genes that are present in the network of gene regulatory interactions. The lasso and ridge regression models were trained using the R *glmnet* package^27^.

**Table 1:** Comparison of classification methods evaluated on simulated and clinical datasets.

#	Method	Network Topology	Upstream Regulators	Description
1	Logistic ridge	−	−	L2-regularized regression^27^
2	Logistic lasso	−	−	L1-regularized regression^27^
3	Filtered gene lasso	−	+	L1-regularized lasso on genes regulated by active regulators.
4	Unweighted group lasso	+	−	Unweighted group lasso^9^ on causal network topology.
5	Sqrt-weighted group lasso	+	−	Group lasso^9^ on causal network topology weighted by sqrt of group size.
6	Regulator-weighted group lasso	+	+	Group lasso with weights by p-values of active regulators
7	Regulator-sqrt-weighted group lasso	+	+	Group lasso with weights by p-values of active regulators and sqrt of group size.

#### Nested cross-validation vs. independent data sets

In all methods under study, internal parameters such as the lasso regularization parameter *λ* in (1) or the tradeoff parameter *α* in (7) must be fit. In addition, we perform a feature selection based on upstream regulator detection prior to classification. The literature abounds with articles in which this situation is inappropriately handled by simple cross-validation. For instance, one could detect transcripts regulated by active regulators on the complete dataset and subsequently employ a cross-validation procedure for the regularized classifier only. This will lead to a strong overestimation of expected classification performance^28^.

The appropriate approach is *nested cross-validation* which wraps feature selection, parameter fitting and classification into nested loops such that no information on the complete dataset is ever used for fitting on the current training slice. In Additional File 1, we outline the employed procedure in more detail. As performance metrics we will focus on balanced accuracy (i.e. the average of sensitivity and specificity) which is an appropriate measure when positive and negative class sizes are not equal. Even appropriate nested cross-validation can, in principle, not account for shifts in the underlying population when the trained classifier is applied in a production setting. Therefore, we chose biological datasets for which we could also evaluate our approaches in two completely independent scenarios.

#### Generation of simulated data

**Figure 2:**
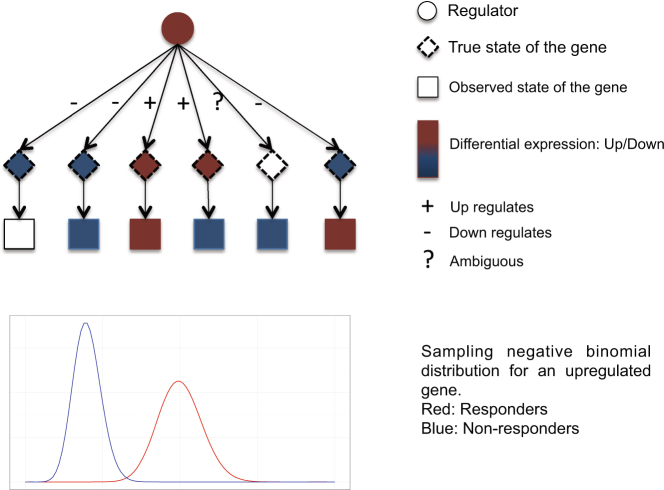
Schematic representation of the simulation process. A Bayesian network is constructed from the causal graph (top) and used as a generative model. Regulators are set to ‘up’ or ‘down’ to distinguish responders and non-responders and the downstream differentially expressed genes are simulated using the Bayesian network. The true (hidden) state of the genes are used to account for noise in gene expression measurements. Two negative binomial models are then utilized to simulate the expression value of up-regulated gene for responders (red curve) and non-responders (blue curve). Expression of down-regulated genes is simulated by switching the mean values. Unregulated genes are generated using a negative binomial model with mean *μ*_0_.

If the difference between responders and non-responders in a given dataset is governed through previously described upstream regulatory mechanisms^29^, our proposed method should perform well. In contrast, on a random network our approach should perform no better than purely statistical regularization approaches such as lasso. To test this hypothesis, we simulated a total of 100 datasets with 1,2,…, 10 randomly selected regulators in the network regarded as ‘active’. Each dataset contains 50 responders and 50 non-responders for training and the same number of samples for testing. The simulation procedure uses a previously described Bayesian network model^15^ to obtain the set of differentially expressed genes given active regulators. Next, we used a negative binomial model to generate gene expression values based on their differential expression status^30^. For unregulated genes, we used a negative binomial model with mean *μ*_0_ and dispersion *r* = *μ*_0_/3. For up-regulated genes, the mean was set to *μ*_1_ = *cμ*_0_ for responders and *μ*_2_ = (1/*c*)*μ*_0_ for non-responders. Here *c* denotes the fold change. The mean values were switched in the case of down regulated genes. In our simulations we used *μ*_0_ = 3 and fold change *c* = (3)^1/2^. The simulated values were then normalized using the R package voom. Figure 2 shows a schematic plot of the simulation process.

#### Processing of clinical data

Clinical trial datasets from (a) acute kidney rejection^31,32^ (GSE50058 and GSE21374) and (b) response to infliximab therapy in ulcerative colitis (UC)^33^ (GSE12251 and GSE14580) were obtained and RMA normalized and differentially expressed genes (DEGs) were identified. All analyses were performed using standard pipelines from the R Bioconductor package^34^. A p-value of 0.05 and a fold change of 1.5 were used as cutoff values in differential gene expression analysis. The DEGs were then used as input to the QuaternaryProd package^16^ to calculate the statistical significance of the upstream regulators. A cutoff of 0.01 was used for the *p*-values to identify significant groups.

## Results

In a series of numerical experiments using artificial and real clinical trial datasets, we tested the ability of our model to predict phenotypic response and to identify relevant associated biomarkers. In the following sections we discuss the results of these experiments.

### Simulations

**Figure 3:**
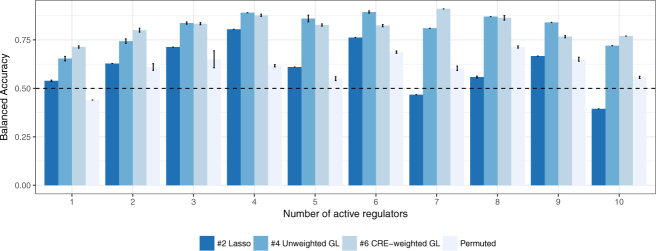
The bar plots show the achieved balanced accuracy in simulated test data for method #6 CRE-weighted group-lasso using the original network and the permuted network in contrast with #2 lasso-based logistic regression as well as #4 Unweighted group-lasso. Across all 10 runs, our method is able to exploit the network-structure to improve performance with respect to lasso-based regression. For a random network, performance deteriorates to the level of lasso-based regression.

Figure 3 shows results of our simulations across the range of simulation parameters. If the network captures the underlying regulatory structure correctly, the proposed creNET method performs well.

### Assessing model performance

**Figure 4:**
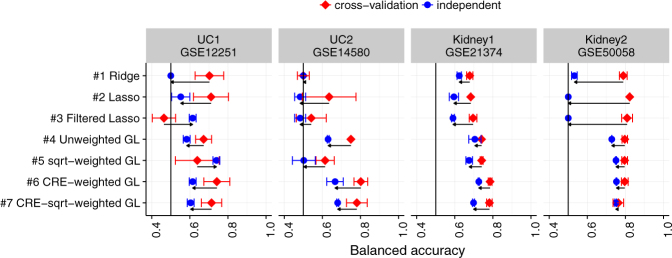
Overview of model performance in nested cross-validation (red diamonds) and independent test sets (blue squares). Each row depicts performance in one of the methods outlined in Table 1. The x-axis corresponds to the average achieved balanced accuracy in multiple runs using the best *λ* value in each run. The horizontal error bars correspond to one standard deviation. Arrows indicate the magnitude of the change between cross-validation and independent tests. Note that in almost all cases performance deteriorates in the independent setting and that prior-knowledge based methods are better able to retain high predictive performance.

Figure 4 shows an overview of performance in terms of balanced accuracy split by cross-validation and independent test set runs. All runs are based on the creKB. First, we focus on balanced accuracy under nested cross-validation, i.e. the expected performance of the trained classifier in a new class-balanced dataset from the same underlying population. Red diamonds in Fig. 4 show that performance of tested method is broadly consistent across the board - with our proposed methods (#6 and #7) showing superior performance in the UC2 and Kidney1 datasets.

As outlined before, we are particularly interested in the robustness of trained classifier when used in independent studies with clinically highly similar phenotypes. Blue circles in Fig. 4 depict balanced accuracy in the corresponding independent datasets. Arrows give a visual indication of the magnitude of the change between cross-validation and independent test set results. In virtually all cases, classifier performance deteriorates when applying the fitted models to the independent datasets with slightly different underlying characteristics. This is especially pronounced for the kidney datasets in which the non-group-lasso methods (#1, #2, and #3) either deteriorate to random predictions (Kidney2 data) or to weak predictivity (Kidney1 data). Notably, our proposed methods show robust performance in all cases.

### Active regulators

**Figure 5:**
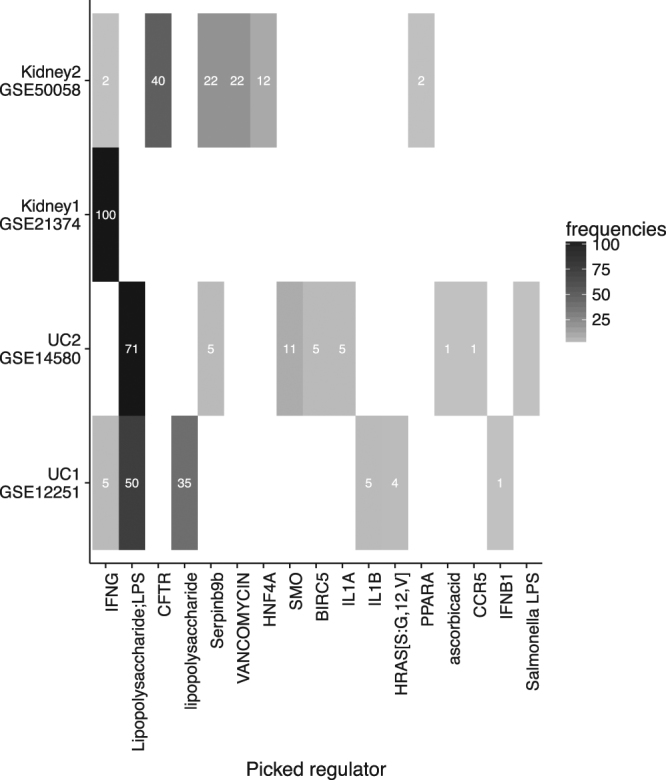
The heatmap shows heterogeneity in picked regulators within and between datasets based on 100 bootstrap runs. Any regulator that was picked at least once in any dataset is displayed. Numbers indicate the number of times a specific regulator was chosen in the 100 bootstrap runs conducted for each dataset.

In addition to its robust performance, creNET facilitates the biological interpretation of the predictive signals by providing ‘regulators’ that can be easily related to the biology that likely drives the response. To further assess the reproducibility and consistency of the selected regulators, we performed a bootstrap analysis as follows. For each dataset, a bootstrap sample was selected and the CRE weighted GL (#6) was trained using cross validation and the regulators selected by the model were recorded. This process was repeated a 100 times and the frequency of every selected predictors was recorded. Figure 5 shows the frequencies of selected regulators in each dataset. Any regulator picked at least once in any dataset is displayed providing an overview of the heterogeneity within and between datasets. For example, in the case of infliximab response in UC, the predominantly selected ‘regulator’ is Lipopolysaccharide;LPS (and its synonym lipopolysaccharide), which is a key activator of the toll-like receptor 4 (TLR4). TLR4 signaling in intestinal epithelial cells in turn is a key component of homeostasis and activation of the TLR4 pathway and has been associated with both Ulcerative colitis and Crohn’s disease^35,36^. The link between infliximab sensitivity and TLR4 is further supported by findings that infliximab induced blockade of TNF-alpha can lead to strong suppression of TLR4 expression^37^. While other components of inflammatory signals may contribute to the overall differential gene expression, creNET directly establishes the hypothesis that differences in the state of TLR4 signaling (via sensitivity to LPS) at baseline may be a key distinguishing factor in the response to infliximab.

In the case of acute kidney rejection, the regulators selected by the algorithm appear at the first glance to be ‘asymmetrical’, i.e. creNET consistently selects Interferon gamma (IFNG) associated transcripts in the GSE21374 cohort, while IFNG is not selected as much in the GSE500058 cohort and combinations of CFTR, Serpinb9b, Vancomycin and HNF4A are selected more frequently instead. However, acute kidney rejection can be mechanistically separated into two distinct concepts: T-cell mediated rejection (TCMR) and antibody mediated rejection (ABMR)^38,39^. IFNG is well documented as a cytokine involved in innate immune response and genes induced by IFNG are shared between TCMR and ABMR^39^. It is therefore intuitive that IFNG regulated transcripts are suitable in predicting rejection. Additionally, a third concept should also be taken into consideration, namely that of acute kidney injury, which appears to be more dominantly represented in the GSE50058 cohort. CFTR, and HFN4A can be interpreted as indicators of kidney function and Vancomycin is well established as a driver for kidney injury. Serpinb9b, the last relevant regulator identified in GSE50058 is again a marker of cytotoxic T-cell activity^40^ and would therefore be consistent with T-cell mediated rejection.

## Conclusions

In this paper we presented a way to integrate biological regulatory networks into a penalized regression and classification framework. The method is specifically designed for phenotype prediction using omics-scale data. We demonstrated that the new penalization and the integration of the relevant biology of gene regulation can lead to more robust predictions that generalize well to independent datasets (Fig. 4). An integral part of the method is the identification of important gene sets defined by the regulators in the network and differential shrinkage of the groups according to their biological relevance (Fig. 5).

Our results show that even when proper care is taken in training and assessing classifiers on high dimensional transcriptomics datasets, models that don’t incorporate prior biological knowledge may still result in poor generalization to independent test data with slightly shifted characteristics. By integrating prior knowledge into the classification framework the model appears to select predictors that are more biologically relevant across a wider, more heterogeneous population.

We provide a user-friendly R-package *creNET* for download from github at https://github.com/kouroshz. The package is accompanied with a parsed version of the STRING DB, but any other regulatory network can be used. Moreover, we provide command-line scripts to facilitate batch runs. Note that performance of the model depends on the assumption that the network captures the underlying biology of the disease. Figures and results have been reproduced using the current freely available version of the STRING DB network^25^ and can be found in the Additional File 1. With the public network we still see equivalent or better performance when compared to the lasso approach in most scenarios, albeit not as strongly as with our proprietary knowledge base. Especially the generalization performance to independent datasets is appreciably weaker using the public network. We regard these results as a call to improve publicly available networks to provide even better substrate for prior-knowledge driven prediction approaches.

As future work we plan the extension of our method to non-linear classification. The described approach should generalize to SVM-based prediction approaches and we hope to further push the boundary for robust classification of phenotypes based on omics data. To summarize, we provide a method that encodes prior biological knowledge of regulatory interactions into the classification process for prediction of clinical phenotypes using baseline or early stage gene expression measurements. This integration results in optimal model sparsity. Our method outputs inferred active upstream regulators, which increases the interpretability of the results.

## Electronic supplementary material

Supplementary Information
