# Supplementary material for: Robust phenotype prediction from gene expression data using differential shrinkage of co-regulated genes
Source: Sci Rep. 2018 Jan 19;8:1237. doi: 10.1038/s41598-018-19635-0 (PMC5775343; doi:10.1038/s41598-018-19635-0)
Supplement: Supplementary file 1 — Supplementary Information [file 41598_2018_19635_MOESM1_ESM.pdf]

Supplementary Information: Robust phenotype prediction from gene expression data using differential shrinkage of co-regulated genes

Kourosh Zarringhalam[1], David Degras[1], Christoph Brockel[2], and Daniel Ziemek[2,*]

[1]Department of Mathematics, University of Massachusetts Boston, Boston MA 02125, USA.
[2]Computational Sciences, Pfizer Worldwide Research & Development, Cambridge MA 02139, USA.
[*]Daniel.Ziemek@pfizer.com

ABSTRACT

Discovery of robust diagnostic or prognostic biomarkers is a key to optimizing therapeutic benefit for select patient cohorts - an idea commonly referred to as *precision medicine*. Most discovery studies to derive such markers from high-dimensional transcriptomics datasets are weakly powered with sample sizes in the tens of patients. Therefore, highly regularized statistical approaches are essential to making generalizable predictions. At the same time, prior knowledge-driven approaches have been successfully applied to the manual interpretation of high-dimensional transcriptomics datasets. In this work, we assess the impact of combining two orthogonal approaches for the discovery of biomarker signatures, namely (1) well-known lasso-based regression approaches and its more recent derivative, the group lasso, and (2) the discovery of significant upstream regulators in literature-derived biological networks. Our method integrates both approaches in a *weighted group-lasso* model and differentially weights gene sets based on inferred active regulatory mechanism. Using nested cross-validation as well as independent clinical datasets, we demonstrate that our approach leads to increased accuracy and generalizable results. We implement our approach in a computationally efficient, user-friendly R package called *creNET*. The package can be downloaded at https://github.com/kouroshz/creNet and is accompanied by a parsed version of the STRING DB data base.

1 Assessing model performance using STRING DB

Figure 1 shows an overview of performance in terms of balanced accuracy split by cross-validation and independent test set runs. All runs are based on the public network STRING DB[1].

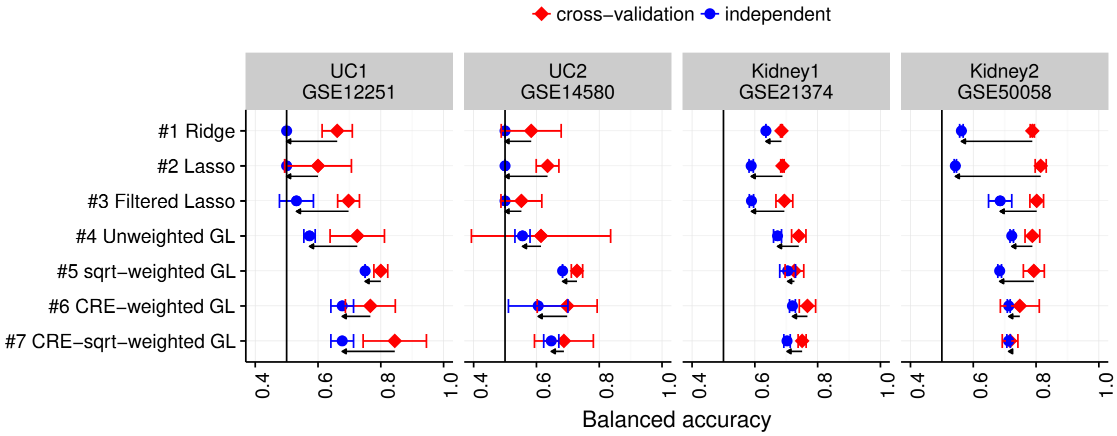

Figure 1. Overview of model performance in nested cross-validation (red diamonds) and independent test sets (blue squares). Each row depicts performance in one of the methods outlined in the main paper. The x-axis corresponds to achieved balanced accuracy. Note that in almost all cases performance deteriorates in the independent setting and that prior-knowledge based methods are better able to retain high predictive performance.

As can be seen, creNET performs generally better than Lasso and seems to keep the consistency across CV and independent test runs. Figure 2 shows a comparison between the results obtained by using the creKB and the publicKB. As can be seen the

results are fairly similar for most datasets.

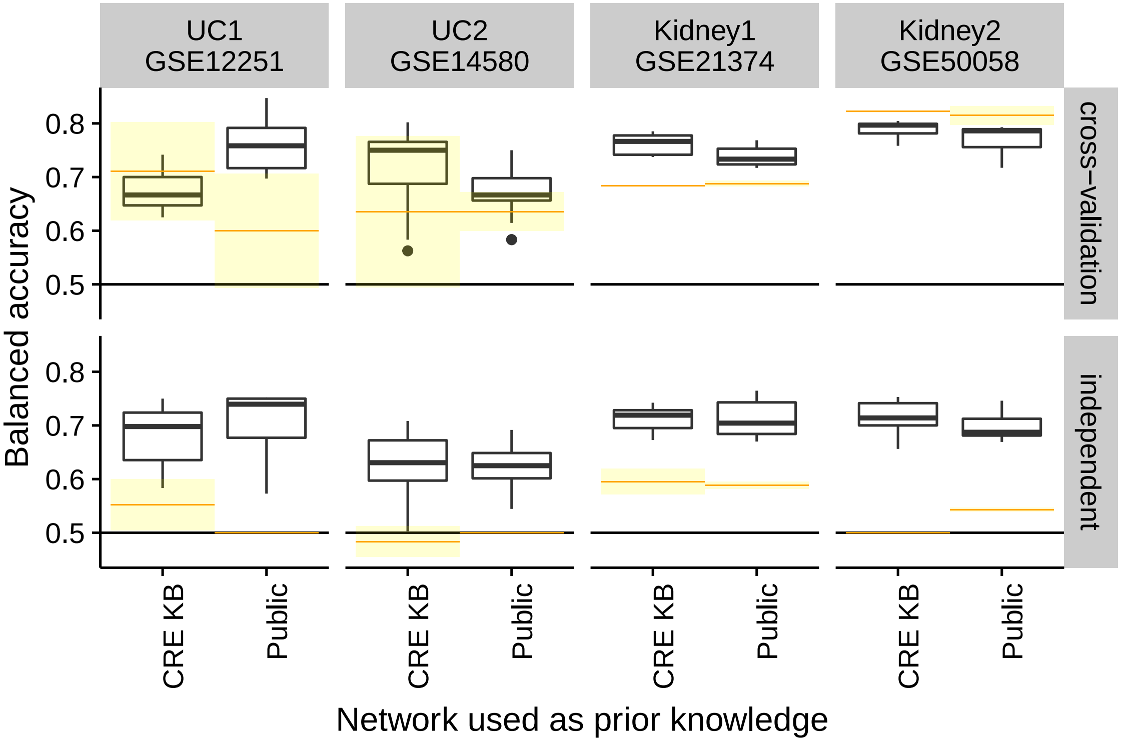

Figure 2. Model comparison between the publicKB and the creKB

1.1 Implementation

Algorithm 1 presents the employed *nested cross validation* procedure for fitting the optimal value of the tuning parameter λ. Training and testing process was carried out according to Algorithm 2. The parameter α represents the tradeoff parameter between group-lasso and lasso (equation 1). Causal Reasoning refers to the algorithm that estimates the statistical significance of upstream regulators as implemented in[2]. Our `R` package *creNET* is available to download from github at https://github.com/kouroshz.

Equivalence of duplicate norm with overlap group lasso

Here we provide a proof for equivalence of overlap group lasso penalty and the duplicated group lasso penalty. Let $M = \sum_{j=1}^{K} \#(g^{(j)})$ be the total number of covariates, taking their multiplicity into consideration. For each group $g^{(j)}$, let $X^{(j)}$ be the $n \times \#(g^{(j)})$ slice of the design matrix X that contains the columns associated with the group $g^{(j)}$, $j = 1, \cdots, K$. Let $\tilde{X} = [X^{(1)}, \cdots, X^{(K)}]$ be the concatenation (duplication) matrix of size $n \times M$ and let $\tilde{\mathbf{x}}_i$, $i = 1, \cdots, n$ denote the rows of $\tilde{X}$. For a vector $\tilde{\beta} \in \mathbb{R}^M$, we write $\tilde{\beta} = (\tilde{v}^{(1)}, \cdots \tilde{v}^{(K)})$ with $\tilde{v}^{(j)} \in \mathbb{R}^{\#(v^{(j)})}$, $j = 1, \cdots, K$. To each $\tilde{v}^{(j)}$, we associate a vector $v^{(j)} = (0, \cdots, 0, \tilde{v}^{(j)}, 0, \cdots, 0) \in \mathbb{R}^p$ such that $\text{supp}(v^{(j)}) \in g^{(j)}$. Define

$$\Phi_1(\beta) = \frac{1}{n} \sum_{i=1}^{n} \Phi(y_i, \beta^T \mathbf{x}_i) + \alpha\lambda ||\beta||_{\mathscr{O}} + (1-\alpha)\lambda ||\beta||_1, \tag{1}$$

and

$$\Phi_2(\tilde{\beta}) = \frac{1}{n} \sum_{i=1}^{n} \Phi(y_i, \tilde{\beta}^T \tilde{\mathbf{x}}_i) + \alpha\lambda ||\tilde{\beta}||_{\mathscr{G}} + (1-\alpha)\lambda ||\tilde{\beta}||_1. \tag{2}$$

Note that $\tilde{\beta} \in \mathbb{R}^M$ and $||\tilde{\beta}||_{\mathscr{G}} = \sum_{j=1}^{K} ||v^{(j)}||_2$.

Input : Training data D, λ-grid $\{\lambda_1, \ldots, \lambda_m\}$, number k of cross-validation folds
Output : Cross-validation performance

Divide the training set $D = (X, Y)$ into $k+1$ random subsets $D_0, \ldots, D_k$ of roughly equal size;

for $o \leftarrow 1$ **to** $k+1$ **do**
  Take D_o as the outer test data D_{outer};
  Take $D \setminus D_o$ as the inner training data D_{inner};
  if *applying group-wise penalty* **then**
    Run causal reasoning on D_{inner} and generate the group weights w_j;
    Optionally filter out groups from D_{inner} and D_{outer} with causal reasoning p-values higher than a user-defined threshold;
  end
  Divide the inner training data into k random folds $D_{inner}^{(1)}, \ldots, D_{inner}^{(k)}$;
  for $i \leftarrow 1$ **to** k **do**
    Take the subset $D_{inner}^{(i)}$ as the inner test data D_{test};
    Take the remaining sets $D_{inner} \setminus D_{inner}^{(i)}$ as the inner training data D_{train};
    for $\lambda \in \{\lambda_1, \cdots \lambda_m\}$ **do**
      Fit the model using λ and D_{train};
      Make a prediction on D_{test} and record the loss;
    end
  end
  Pick the λ value that optimizes the loss;
  Fit model using the entire D_{inner} and the optimal λ;
  Perform a prediction on the outer data D_{outer};
end
Compare the prediction results against the true labels;

Algorithm 1: Nested Cross-Validation

Theorem 1. *The functions Φ_1 and Φ_2 have the same minimum*

$$\min_{\beta} \Phi_1(\beta) = \min_{\tilde{\beta}} \Phi_2(\tilde{\beta}).$$

Moreover, the minimizers of Φ_1 can be deduced from those of Φ_2 and vice-versa. More precisely, let $\beta \in \mathbb{R}^p$ be a minimizer of Φ_1 and let $(v^{(1)}, \cdots, v^{(K)})$ be the decomposition of β that minimizes $||\beta||_{\mathscr{O}}$. For each $j = 1, \cdots, K$, let $\tilde{v}^{(j)}$ be the subvector of $v^{(j)}$ indexed by $g^{(j)}$. Then the vector $\tilde{\beta} = (\tilde{v}^{(1)}, \cdots, \tilde{v}^{(K)})$ minimizes Φ_2. Conversely, given a minimizer $\tilde{\beta} = (\tilde{v}^{(1)}, \cdots, \tilde{v}^{(K)}) \in \mathbb{R}^M$ of Φ_2 and the associated vectors $v^{(j)} = (0, \cdots, 0, \tilde{v}^{(j)}, 0, \cdots, 0) \in \mathbb{R}^p$, the vector $\beta = \sum_{j=1}^{K} v^{(j)}$ minimizes Φ_1.

Proof. The objective functions Φ_1 and Φ_2 are non-negative, continuous on their respective domains $\mathbb{R}^p$ and $\mathbb{R}^M$, and $\lim_{\|\beta\| \to +\infty} \Phi_1(\beta) = \lim_{\|\tilde{\beta}\| \to +\infty} \Phi_2(\tilde{\beta}) = +\infty$. These conditions guarantee the existence of minima for Φ_1 and Φ_2.

With the notations of Theorem 1, for a given $\beta \in \mathbb{R}^p$, let $(v_1, \ldots, v_K)$ be a decomposition of β such that $\|\beta\|_{\mathscr{O}} = \sum_{j=1}^{K} \|v_j\|_2$ and let $\tilde{\beta} = (\tilde{v}^{(1)}, \cdots, \tilde{v}^{(K)})$ be the associated vector in $\in \mathbb{R}^M$. By construction,

$$\begin{aligned} \|y - X\beta\|_2^2 &= \left\| y - X \sum_{j=1}^{K} v_j \right\|_2^2 \\ &= \left\| y - \sum_{j=1}^{K} X^{(j)} \tilde{v}_j \right\|_2^2 \\ &= \left\| y - \widetilde{X}\tilde{\beta} \right\|_2^2. \end{aligned} \tag{3}$$

It is also clear that

$$\|\beta\|_{\mathscr{O}} = \sum_{j=1}^{m} \|v_j\|_2 = \sum_{j=1}^{m} \|\tilde{v}_j\|_2 = \left\|\tilde{\beta}\right\|_{\mathscr{G}}. \tag{4}$$

Input : Training data; Testing data (optional) and the Network
Output : Cross-validation and/or independent train and test performance with selected predictors

If testing data is provided match the train and test data so that the columns of the input matrices represent the same covariates in exactly the same order;
Process the data to match the genes to the network; filter out the genes not present in the network and identify the groups;
Normalize the training and testing data if needed;
for $\alpha \in \{\alpha_1, \cdots, \alpha_k\}$ **do**
    if *test data is provided* **then**
        Run causal reasoning on the training data and generate the desired weight of each group;
        Optionally filter out the groups from training and testing data with causal reasoning p-values higher than a user-defined threshold;
        Fit the model using the training data and select the optimal λ value by cross-validation;
        Make a prediction on the test dataset;
    end
    else
        Perform Algorithm 1 (nested cross-validation) on the training data;
    end
end
Select the best α based on cross-validation performance on the training data;
Report the performance results and the nonzero groups and covariates;

Algorithm 2: Main Algorithm

For $i = 1, \ldots, p$, let $\tilde{\beta}_{i1}, \ldots, \tilde{\beta}_{if_i}$ be the coefficients of $\tilde{\beta}$ associated with β_i, where f_i is the frequency of index i in the groups $g_1, \ldots, g_K$. In other words, for all group g_j such that $i \in g_j$, $\tilde{\beta}_{ij}$ is the i-th coefficient of v_j. By definition, $\sum_{k=1}^{f_i} \tilde{\beta}_{ik} = \beta_i$. Furthermore, the $\tilde{\beta}_{ik}$ are either of the same sign as β_i or equal to zero. Indeed, if $\beta_i = 0$, then the $\tilde{\beta}_{ik}$ are also zero so as to minimize the contribution of the v_j ($i \in g_j$) to $\|\beta\|_{\mathscr{O}}$. Similarly, if $\beta_i \neq 0$, say $\beta_i > 0$, and $\tilde{\beta}_{ik_-} < 0$ for some $1 \leq k_- \leq f_i$, then there exists at least one coefficient $\tilde{\beta}_{ik_+} > 0$, otherwise the equality $\sum_{k=1}^{f_i} \tilde{\beta}_{ik} = \beta_i$ would be violated. But by replacing $\tilde{\beta}_{ik_+}$ with $(\tilde{\beta}_{ik_+} + \tilde{\beta}_{ik_-})_+$ in v_{k_+} and $\tilde{\beta}_{ik_-}$ with $(\tilde{\beta}_{ik_+} + \tilde{\beta}_{ik_-})_-$ in v_k, one would obtain new vectors $v_1^*, \ldots, v_K^*$ such that $\sum_j v_j^* = \beta$, $\mathrm{supp}(v_j^*) \subset g_j$ for all j, and $\sum_j \|v_j^*\|_2 < \sum_j \|v_j\|_2 = \|\beta\|_{\mathscr{O}}$, a contradiction. The case where $\beta_i < 0$ can be treated in the same way. In light of the fact that $\tilde{\beta}_{i1}, \ldots, \tilde{\beta}_{if_i}$ are either of the same sign as β_i or equal to zero for all $i = 1, \ldots, p$, it is straightforward to see that

$$\|\tilde{\beta}\|_1 = \sum_{i=1}^{p} \sum_{k=1}^{f_i} |\tilde{\beta}_{ik}| = \sum_{i=1}^{p} |\beta_i| = \|\beta\|_1 . \tag{5}$$

It follows from equations (3), (4), and (5) that for all $\beta \in \mathbb{R}^p$, there exists a $\tilde{\beta} \in \mathbb{R}^M$ such that $\Phi_1(\beta) = \Phi_2(\tilde{\beta})$ and hence

$$\min_{\tilde{\beta} \in \mathbb{R}^M} \Phi_2(\tilde{\beta}) \leq \min_{\beta \in \mathbb{R}^p} \Phi_1(\beta). \tag{6}$$

Consider now a vector $\tilde{\beta} \in \mathbb{R}^M$. Define the associated vectors $v^{(j)}, j = 1, \ldots, K$, and $\beta = \sum_{j=1}^{K} v^{(j)}$ as in Theorem 1. Using the same arguments as before, we can prove that $\|y - X\beta\|_2^2 = \left\|y - \widetilde{X}\tilde{\beta}\right\|_2^2$. In addition, $\left\|\tilde{\beta}\right\|_{\mathscr{G}} = \sum_{j=1}^{K} \left\|v^{(j)}\right\|_2 \geq \|\beta\|_{\mathscr{O}}$ and $\left\|\tilde{\beta}\right\|_1 = \sum_{j=1}^{m} \|v^{(j)}\|_1 = \left\|\sum_{j=1}^{K} v^{(j)}\right\|_1 = \|\beta\|_1$ by definition of β and $\|\cdot\|_{\mathscr{O}}$. Combining these facts, we obtain:

$$\min_{\tilde{\beta} \in \mathbb{R}^M} \Phi_2(\tilde{\beta}) \geq \min_{\beta \in \mathbb{R}^p} \Phi_1(\beta). \tag{7}$$

Gathering (6) and (7), the first part of the theorem is proved. The second part of the theorem immediately follows from the first part and the facts that: (i) for a given $\beta \in \mathbb{R}^p$, the associated $\tilde{\beta} \in \mathbb{R}^M$ satisfies $\Phi_2(\tilde{\beta}) = \Phi_1(\beta)$, and (ii) for a given $\tilde{\beta} \in \mathbb{R}^M$, the associated $\beta \in \mathbb{R}^p$ satisfies $\Phi_1(\beta) \leq \Phi_2(\tilde{\beta})$.

□

References

1. Szklarczyk, D. *et al.* String v10: protein–protein interaction networks, integrated over the tree of life. *Nucleic acids research* gku1003 (2014).
2. Fakhry, C. T. *et al.* Interpreting transcriptional changes using causal graphs: new methods and their practical utility on public networks. *BMC bioinformatics* **17**, 318 (2016).
